# Full-Field Measurements in the Edge Crush Test of a Corrugated Board—Analytical and Numerical Predictive Models

**DOI:** 10.3390/ma14112840

**Published:** 2021-05-26

**Authors:** Tomasz Garbowski, Jakub Krzysztof Grabski, Aleksander Marek

**Affiliations:** 1 Department of Biosystems Engineering, Poznan University of Life Sciences, Wojska Polskiego 50, 60-627 Poznań, Poland; tomasz.garbowski@up.poznan.pl; 2 Institute of Applied Mechanics, Poznan University of Technology, Jana Pawła II 24, 60-965 Poznań, Poland; 3 Faculty of Engineering and Physical Sciences, University of Southampton, Highfield SO171BJ, UK; a.marek@soton.ac.uk

**Keywords:** corrugated cardboard, edge crush test, orthotropic elasticity, digital image correlation

## Abstract

This article focuses on the derivation of simplified predictive models for the identification of the overall compressive stiffness and strength of corrugated cardboards. As a representative example an unsymmetrical 5-ply sample (with E and B flute) was used in this study. In order to exclude unreliable displacement measurement in the standard edge crush test, virtual strain gauges were used. Video extensometry was employed to collect measurements from the outer surfaces of the sample on both sides. Additional data allowed real force-displacement curves to be obtained, which were used in the validation procedure. To emulate the experimental results, besides a simple analytical model, a 3D numerical model fully reflecting the geometry of the corrugated board, based on the finite elements method was also built. In both cases good agreement between the experimental results and the analytical and numerical calculations was observed. This proved that the proposed analytical model can be successfully used to determine the overall stiffness and compressive strength of corrugated board, provided that the geometry and properties of all the layers of the board are known. The simple model presented in this work enables quick and reliable design and prototyping of new assemblies without the need to manufacture them.

## 1. Introduction

Prediction of material strength is an important issue for designing and manufacturing of products made from corrugated paperboard. In the literature, authors have applied many different approaches for strength investigations of corrugated sandwich structures, including paperboard, i.e., analytical [1,2], numerical [3,4,5,6,7], or analytical-numerical [8,9,10] methods. Recently, Kmita-Fudalej et al. presented an analytical prediction of the strength of honeycomb paperboard based on the mechanical properties of the paper used and the geometrical features of the investigated structure [11]. Park et al. performed numerical simulation using the finite element method (FEM) in order to estimate the strength in the edge crush test (ECT) [12]. Recently, artificial intelligence methods have become popular, e.g., artificial neural networks, for prediction of strength of composite materials, including sandwich structures [13]. An alternative to the numerical prediction of the strength of corrugated board is its experimental measurement.

To perform numerical simulations, detailed knowledge of the material properties of the constituents is required. This is however a challenging task, due to the inherent anisotropy of paper-based materials. As a result, physical testing of corrugated paperboard is much more popular within the industry.

A number of typical tests to characterize mechanical properties of corrugated paperboard have been developed to standardize the process. The compressive strength is investigated by performing the ECT, in which the loading is applied perpendicularly to the axis of the flutes. In the bending test (BNT), four-point bending test is performed, in which there are two supports at the bottom of the paperboard and two equal forces acting on the sample from the opposite side. The shear stiffness test (SST) is carried out by applying a pair of forces on the opposite corners (two others are supported), causing the cross-section of the paperboard to be twisted. The torsional stiffness test (TST) is conducted by twisting a sample in both directions. Other tests of the paperboard are namely bursting and humidity tests. In order to investigate the strength of the whole container made from the corrugated paperboard, the box compressive test (BCT) is carried out [14].

Analytical and numerical predictive models of the strength obtained in the ECT are considered in this paper. The ECT is standardized; there are four different methods of the ECT described in the standards. One of the main features that distinguish these tests is the shape of the specimens. These methods are as follows: edge-clamping method [15], neck-down method [16], rectangular test specimen method [16,17,18], and edge-reinforced method [19,20]. Here, the rectangular test specimen method with a specimen with dimensions of 100 mm × 25 mm was used. More details about these standards of the ECT can be found in [21].

As a verification method for the results obtained from analytical and numerical approaches, presented in this study, a video extensometry technique was used, where pairs of points are tracked across images taken at various levels of loading and their relative distance is measured. This is a similar, but simpler approach to digital image correlation (DIC), which is an advanced full-field non-contact optical method of measurement that is recently becoming popular in the area of experimental mechanics, due to its very high accuracy. However, application of those techniques for investigation of the paperboard strength is rather limited in the literature. Hägglund et al. investigated thickness changes during the ECT in the corrugated paperboard using the DIC [22]. The authors examined both damage and undamaged panels. In the series of papers [23,24,25], Viguié and collaborators employed the DIC technique in order to study the strain and stress fields of paperboard panels during the box compression test. Borgqvist et al. proposed a distortional hardening plasticity model for paperboard [26]. The authors introduced a yield surface described by multiple hardening variables and showed that they can be obtained from simple uniaxial experiments. The results obtained from the model were compared with the results obtained from experiments using DIC. Cocchetti et al. investigated identification of material parameters of anisotropic elastic-plastic material models in the case of foils [27,28]. The authors considered paperboards and laminates for liquid containers. They performed combined compression and bending tests using DIC. On the other hand, numerical simulations using the FEM were used in a direct analysis. The parameters of the model were obtained from an inverse analysis, employing results of the experiment and simulations. Considine used DIC and the virtual fields method (VFM) technique to identify general anisotropy parameters of a filter paper and a paperboard [29]. Åslund and collaborators investigated the failure mechanism of the corrugated sandwich panels during the ECT using the detailed finite element method and compared it with the measurements obtained using DIC [30]. Zappa et al. investigated inflation of the paperboard composites using in beverage packaging using the DIC technique [31]. Recently, Fadiji et al. employed DIC to analyze a paperboard box with ventilation holes under compression loading [32]. In most of the investigations mentioned above, samples of 3-ply corrugated cardboards were examined. In this study, an optical method was employed to verify the analytical and numerical results in the ECT analysis of double-wall corrugated cardboard, i.e., 5-ply corrugated cardboard samples with E and B flutes.

Here, analytical and numerical models are proposed to identify paperboard stiffness and to predict the compressive strength of the corrugated paperboard. Optical extensometry is employed to validate the obtained results. Both the analytical and numerical approaches achieved accurate results.

## 2. Materials and Methods

### 2.1. Corugated Cardboard

**Figure 1:**
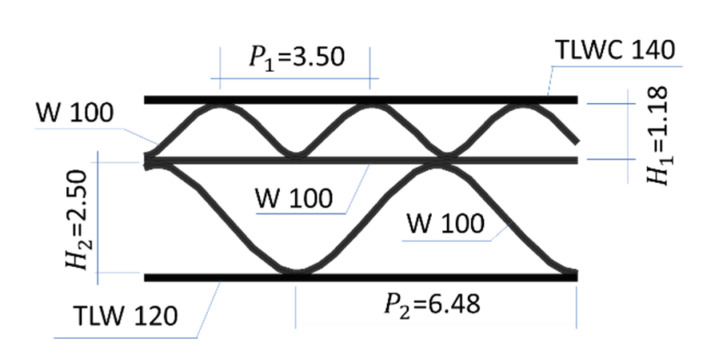
The cross section of 5EB650C3 corrugated board.

In the research, 5-layer corrugated cardboard named 5EB650C3, produced in Aquila Września—the Polish branch of the VPK Group—was used. This grade consists of an external coated layer of a white recycled base liner board with a grammage of 140 g/m^2^. Both corrugated layers (E and B flutes) and the flat layer in between are made from lightweight recycled fluting WB with a grammage of 100 g/m^2^. As an internal layer again the white test liner with a grammage of 120 g/m^2^ was used. The arrangement of individual layers and the geometry of the cardboard cross-section are shown in Figure 1.

The geometrical features of both corrugated layers (flutes) are presented in Table 1. Take-up ratio is defined as the ratio of the length of the non-fluted corrugated medium to the length of the fluted web. For the correct numerical modeling of corrugated layers, a sine-shaped corrugated layer is usually considered. This however is an approximation to the real shape of the flute produced. The theoretical take-up factors can be computed from the formula:

(1) $$\alpha =\frac{1}{P}\int_{O}^{P}\sqrt{1+{\left(\pi \frac{H}{P}\cos\left(\frac{2\pi x}{P}\right)\right)}^{2}}dx,$$

where H is the height, P denotes the pitch. Thus, for the E-flute one can obtain α=1.239, and for the B-flute, α=1.302, which are very close to the actual values given in Table 1. The above formula results from a sine-like shape assumption and is equal to the length of the fluting divided by the flute pitch (wave period).

**Figure 2:**
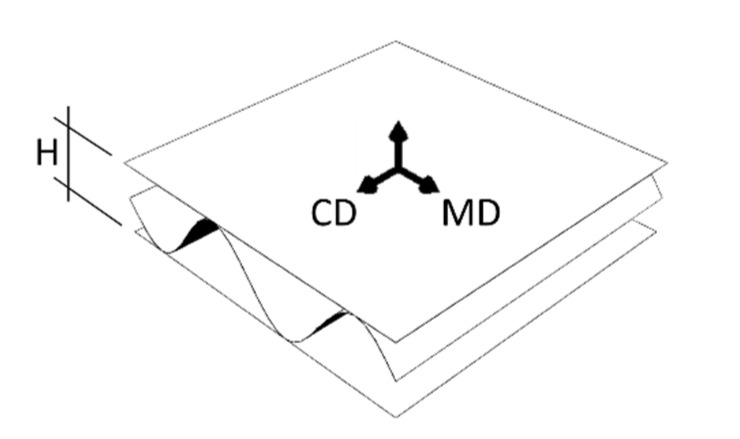
The material orientation in corrugated board.

Since corrugated cardboard consists of several layers of paperboard, made of cellulose fibers, its mechanical properties depend on the fiber orientation of its components. In paperboard, two main, mutually perpendicular directions can be determined. First, along the fiber orientation, which is called Machine Direction (MD). Material is both stiffer and stronger in this direction. The second is perpendicular to the MD and is called Cross Direction (CD). The paper-forming fibers make the corrugated board also an orthotropic material, in which the MD is along the waves (see Figure 2). The corrugated layers thus compensate through take-up factor for the weaker mechanical performance of the cardboard in the CD.

Specified by the producer, the compressive strength, ECT of the combined corrugated board (5EB650C3) in CD is 7.6 kN/m (±10%), while its overall thickness H is 4.3 mm (±0.2 mm).

The material properties of the individual layers are presented in Table 2. The $SCT_{CD}$ value represents a compressive strength in CD from the short-span compression test according to DIN EN ISO 3037 [18].

### 2.2. Measurements

**Figure 3:**
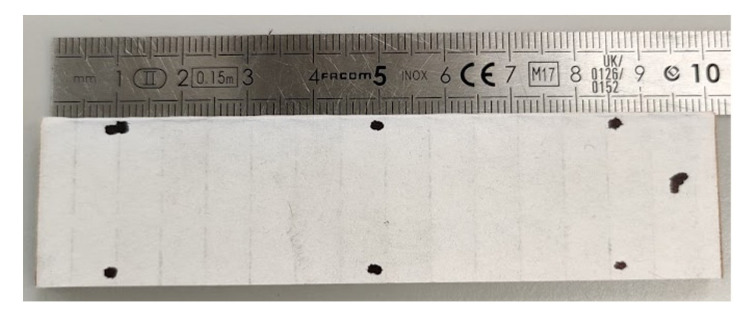
The sample for the edge crush test.

**Figure 4:**
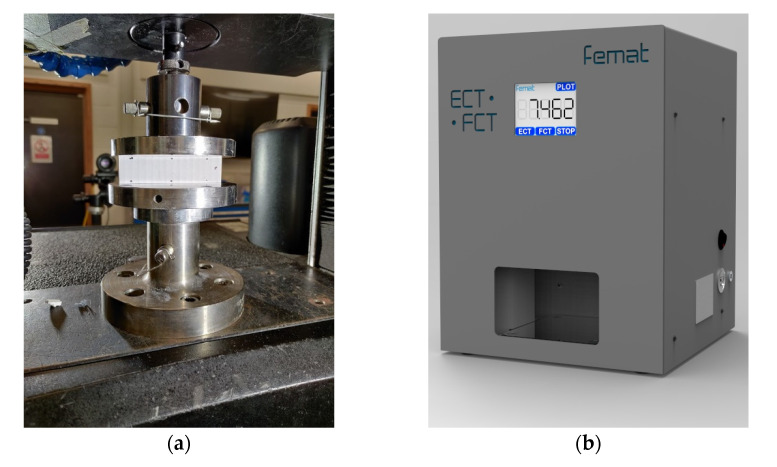
Edge crush test: (**a**) Universal Testing Machine (Instron 5569); (**b**) FEMAT lab. device.

A typical test to determine the compressive strength of corrugated board is the ECT (according to the FEFCO standard DIN EN ISO 3037 [17,18]), in which a specimen that is 100 mm long and 25 mm high (see Figure 3) is loaded along its height between two rigid plates (see Figure 4a). The samples should be cut on a special cutter with the use of one-sided ground blades to maintain the parallelism of the cut edges. According to the standard, the air condition should be controlled, and the test should be carried out at 23 °C and 50% relative humidity. All the ECT tests were performed in a controlled environment as standard on an FEMat ECT/FCT laboratory apparatus (FEMat Sp. z o. o., Poznan, Poland) [33], see Figure 4b.

The ECT is used explicitly to determine the compressive strength of the corrugated board in CD. Although most testing machines allow the recording of curves from the entire test, it is not possible to use these curves for a reliable determination of e.g., compression stiffness. The measured displacements do not represent the elastic deformation of the specimen as they are significantly affected by the clearance and susceptibility on the crosshead, local pressure on sample unevenness (edge effects), etc. Therefore, non-contact optical techniques are required to reliably measure displacements (deformations or strains). Additionally a measure without direct contact does not influence the measure. In measurements with contact (e.g., traditional extensometers), noise is introduced into the measurement and thus the actual measured values are distorted.

### 2.3. Optical Measurements of Sample Deformation

**Figure 5:**
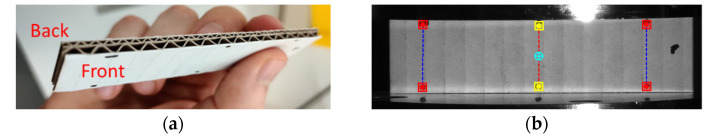
Specimen: (**a**) back and front face of the specimen; (**b**) virtual extensometers on the front face of the specimen.

In this study, the specimen was also tested using optical extensometry. Two cameras were used to track the deformation of both faces to account for the out-of-plane bending produced by the non-symmetrical section. The front face is the higher flute, while the back face is the lower flute of the paperboard, see Figure 5a. Each of the two faces of the specimen was marked with three sets of dots in order to enable point tracking. In Figure 5b, one can observe the sets of points on one of these faces. The single set of points, marked in this figure by squares connected by a dotted line, is a virtual extensometer, for which the extension is observed during the test. The video extensometry was performed using MatchID DIC platform (v. 2020.2.0, MatchID, Ghent, Belgium). The specimen was sandwiched between two platens and aligned using 3D printed L-brackets.

**Figure 6:**
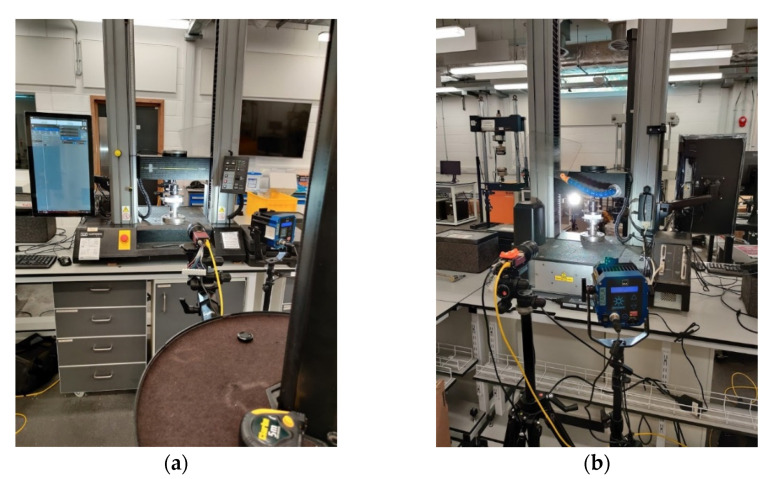
Setup of the optical measurements: (**a**) camera recording the front face; (**b**) camera recording the back face.

Two 5 MPix cameras (Manta G504-b, Allied Vision, Allied Vision, Stadtroda, Germany) were used to record grey scale images during the test, see Figure 6. Cameras were calibrated using MatchID calibration plate (MatchID, Ghent, Belgium) to obtain the pixel to mm conversion rate of ~50 µm/pix. The specimen was manually pre-loaded to a very small load (15 N) to make sure both plates were in contact. After that the measured load cell and displacement were zeroed and the supporting L-brackets removed. Once the cameras started recording, the sample was loaded using displacement control at 0.5 mm/min. The load and the crosshead displacement were synchronized with the cameras. The virtual extensometers were used to measure displacement between the marked points. They were placed roughly 2 mm away from the loading edge in order to avoid measuring additional phenomena occurring in the surrounds of the loading edge. The accuracy of the measurement was estimated using a set of 25 static images (without any movement); standard deviation of the measured elongation was e4valuated to be 4 µm, which can be considered the level of uncertainty. Optical displacements were averaged for each face and compared against the crosshead displacement.

### 2.4. Predictive Models

Two different models were used to estimate the compressive strength of the corrugated board in the CD: (a) a simplified analytical model and (b) a fully detailed 3D numerical model. The former model is based on an iterative procedure, while the latter model is based on the FEM.

**Figure 7:**
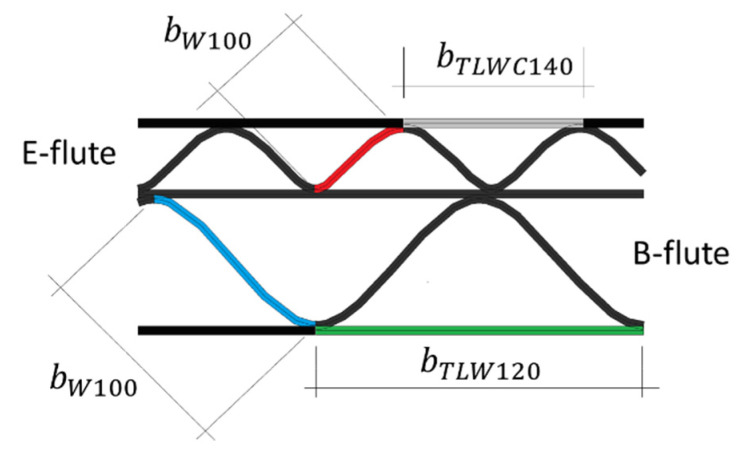
Width of the *i*-th layer.

The simplified estimation procedure proposed here consists of a simple analytical model and uses the basic constitutive parameters of the individual *i*-th layer, namely: $SCT_{CD}^{i}$, compressive strength in CD and ${\bar{E}}_{CD}^{i}$, stiffness index in CD. As in some cases single layer instability may occur before plasticity activation, the critical load should be calculated from the formula [8,9,10]:

(2) $$P_{cr}^{i}=\frac{\pi^{2}}{b_{i}^{2}}\frac{t_{i}^{2}}{12}\sqrt{E_{CD}^{i}E_{MD}^{i}}{\left(\frac{mb_{i}}{L}+\frac{L}{mb_{i}}\right)}^{2},$$

where, $b_{i}$ is the width of the separated plate and is related to a pitch or a half-wave length of the flute (see Figure 7); $t_{i}$ is the *i*-th board thickness; $E_{CD}^{i}$ is the stiffness index in CD; $E_{MD}^{i}$ is the stiffness index in MD; L is the sample height (always equal 25 mm); m is the number of half-waves for which $P_{cr}^{i}$ reaches the minimum.

**Figure 8:**
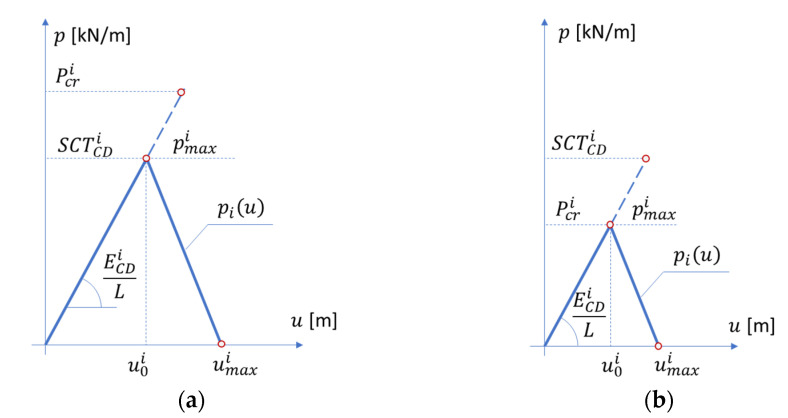
Compressive strength vs. deformation. (**a**) the case where the SCT is lower than the critical load of the *i*-th layer; (**b**) the case where the critical load is lower than the SCT of the *i*-th layer.

The deformation corresponding to the maximum load can be calculated from Hooke’s law considering the stiffness in the CD direction, sample height, L, and the compressive strength or critical load, whichever occurs first (see Figure 8). So for the *i*-th layer the relation takes the form:

(3) $$u_{0}^{i}=\frac{p_{max}^{i}}{E_{CD}^{i}}L,$$

where:

(4) $$p_{max}^{i}=\min\left(SCT_{CD}^{i},P_{cr}^{i}\right).$$

If it is assumed that the failure once initiated successively progresses over time and, for example, for the value of $u_{max}^{i}$, the compression resistance of the *i*-th layer reaches zero, we obtain a bilinear curve describing the constitutive behavior of a single panel. It was assumed on the basis of experimental observations, that the ultimate deformation equals:

(5) $$u_{max}^{i}=\frac{3}{2}u_{0}^{i}.$$

Now, the ECT value can be obtained by simple summation over all layers including the take-up ratio. The displacement-dependent formula for ECT is therefore:

(6) $$ECT\left(u\right)=\overset{n}{\underset{i=1}{\sum }}p_{i}\left(u\right)\;\alpha_{i},$$

where $\alpha_{i}$ is the take-up factor of the corrugated layers calculated by Equation (1) or taken from Table 1.

The second model was built in the Abaqus Unified FEA^®^ [34] software (version 2020, Dassault Systemes SIMULIA Corp., Johnston, IA, USA), which uses a linear elastic orthotropic material model with von Mises plasticity. Shell elements used in the calculations are quadrilaterals with four nodes, named S4, which use the full integration scheme with built-in techniques to prevent locking phenomena. The approximate size of a single element was 1 mm, which gives in total 17,825 elements, 18,668 nodes, and 112,008 degrees of freedom. In order to provide all the required material constants, the empirical equations provided by Baum [35] were used. First the $E_{MD}^{i}$ and $E_{CD}^{i}$ stiffness indexes (given in Table 2) were transformed to stiffness coefficients $E_{1}^{i}$ and $E_{2}^{i}$, respectively, by the equation:

(7) $$E_{1}^{i}=\frac{E_{MD}^{i}}{t_{i}},\;\;\;\;\;E_{2}^{i}=\frac{E_{CD}^{i}}{t_{i}}.$$

The in-plane shear stiffness can be computed from the empirical formula [35]:

(8) $$G_{12}^{i}=0.387\sqrt{E_{1}^{i}E_{2}^{i}}.$$

The Poisson ratio in the 1–2 plane can be assumed from [35] as:

(9) $$\nu_{12}^{i}=0.293\sqrt{\frac{E_{1}^{i}}{E_{2}^{i}}}.$$

Both transversal stiffnesses were computed using the approximation from [36]:

(10) $$G_{13}^{i}=\frac{E_{1}^{i}}{55},\;\;\;\;\;G_{23}^{i}=\frac{E_{2}^{i}}{35}.$$

The compressive strength can be determined by dividing the SCT value in the CD by the appropriate thickness of a single *i*-th layer.

(11) $$\sigma_{0}^{i}=\frac{SCT_{CD}^{i}}{t_{i}}.$$

All the computed values of the constitutive parameters for each layer are summarized in Table 3.

## 3. Results

### 3.1. Edge Crush Test Results

Here, first the results of the edge crush tests are presented. The dispersion of the obtained results is due to the heterogeneity of the corrugated cardboard samples, including local imperfections, lack of parallelism of the sample edges, local detachment of the corrugated layers, etc. Although the specimen is held by steel blocks during the test to prevent global out-of-plane buckling, local buckling on the outer surfaces of the specimen still could be observed. A slight bend, which is the result of the nonsymmetric cross-section of the sample, also could be observed.

**Figure 9:**
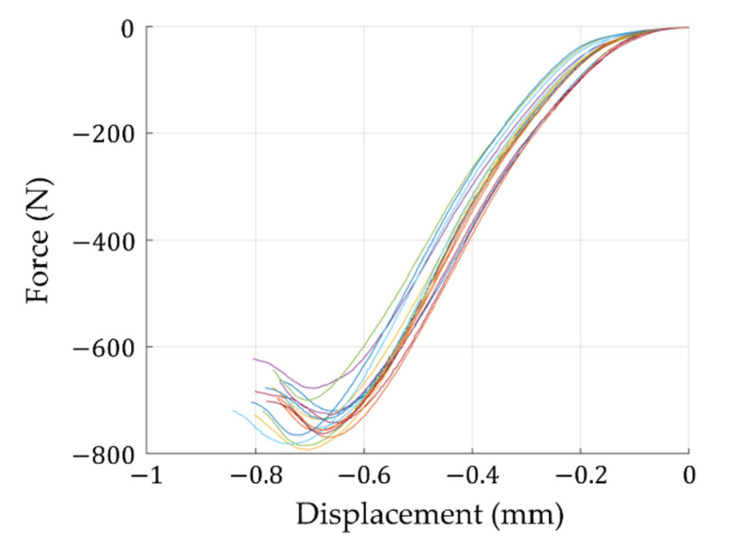
Edge crush test results (on the FEMat ECT/FCT laboratory apparatus).

It is worth noting that the elastic stiffness, which could be determined from the linear part of the experimental curves, is not the real stiffness because it includes all the effects of the crossbar compliance and the sample imperfections, especially visible in the initial part of the curves (see Figure 9).

### 3.2. Optical Measurements Results

**Figure 10:**
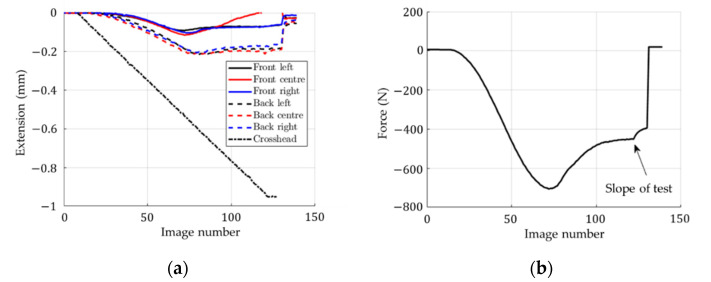
Optical measurements results: (**a**) extension, (**b**) applied force.

Figure 10 presents the results obtained from the video extensometry measurements. In Figure 10a, one can observe the extension in terms of the image number from the virtual extensometers on the front face (on the left side, at the center and on the right side), on the back face (on the left side, at the center and on the right side) and on the crosshead. In Figure 10b, the applied force is shown in terms of the image number. The maximum absolute value of the applied force was approximately 703 N, while the mean value obtained from the ECT measurements was equal to 751 N. However, it should be noted that a pre-load of 15 N was applied and after that the measurements started from zero value. Here, the loading rate (0.5 mm/min) was significantly slower than the typical 10 mm/min due to the limited frame rate of the cameras, which reduces the measured maximum load through relaxation.

### 3.3. Predictive Analytical Model

In the predictive analytical model, constitutive curves are first constructed based on the specific material parameters of the individual layers (see Table 2), based on Equations (2)–(6). The results of the buckling analysis and other parameters necessary to build the constitutive curves are summarized in Table 4.

**Figure 11:**
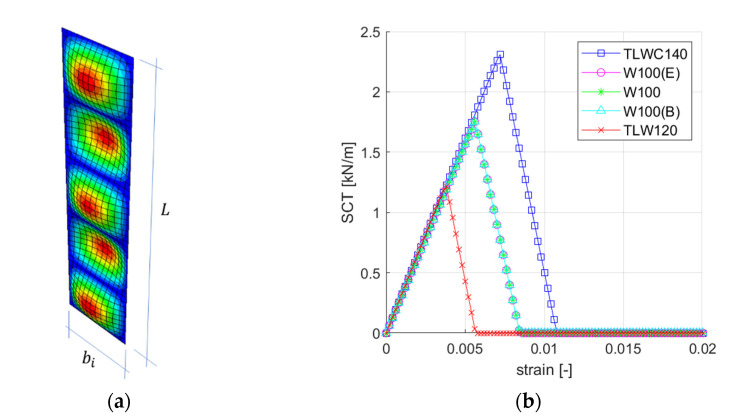
Analytical model: (**a**) Visualization of first buckling mode for the *i*-th layer; (**b**) constitutive relationships for all corrugated board layers.

Figure 11a shows an example of the eigenmode of the individual separated *i*-th plate, calculated as simply supported plate loaded along the L dimension. Figure 11b shows all constitutive curves, where the maximum value of the compressive load is equal to $SCT_{CD}$ for the TLWC140 layer and all W100 layers, while for the TLW120 layer it is the critical load value due to the dimension $b_{i}$ (see Equation (2)), which is the largest in this case.

### 3.4. Predictive Numerical Model

In order to correctly calculate the compressive strength using the FEM in the simulation of the ECT, two steps of the numerical procedure had to be used, namely: (1) perturbation analysis, where the eigenmode and eigenvector were calculated, and (2) geometric and material nonlinear iterative analysis, in which geometric imperfections are introduced based on the calculated eigenvalues and eigenvectors from the first analysis.

**Figure 12:**
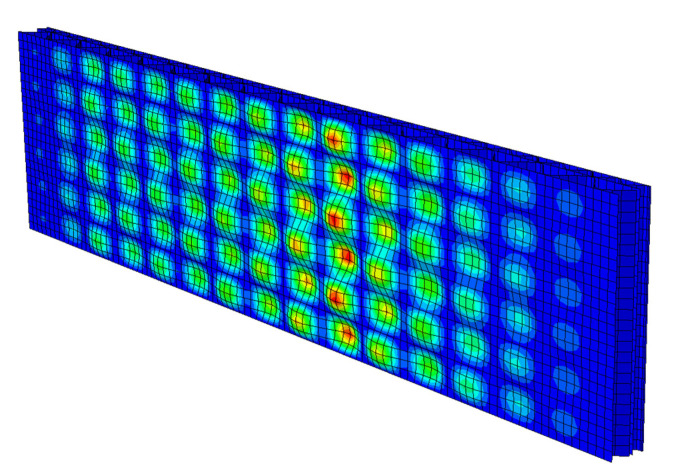
Numerical model. Visualization of the first buckling mode for the whole corrugated board (front view).

The first perturbation analysis was only to find the initial shape imperfection in the numerical model of the ECT sample, which was later entered as the first scaled eigenvector of the model (see Figure 12) in the Abaqus Unified FEA^®^ software. This imperfect geometry was used in nonlinear analysis where the standard Newton–Raphson algorithm was used to find convergence in the subsequent iterations.

**Figure 13:**
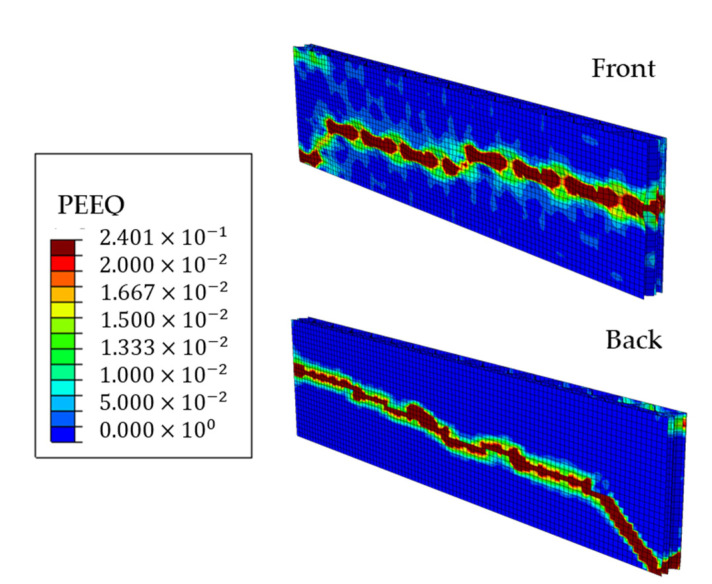
Equivalent plastic strains on both sides of the ECT sample in the last iteration of the nonlinear analysis.

The equivalent plastic strains on both sides of the ECT sample model in the last iteration are shown in Figure 13, where the plasticized region is marked in a dark red color.

**Figure 14:**
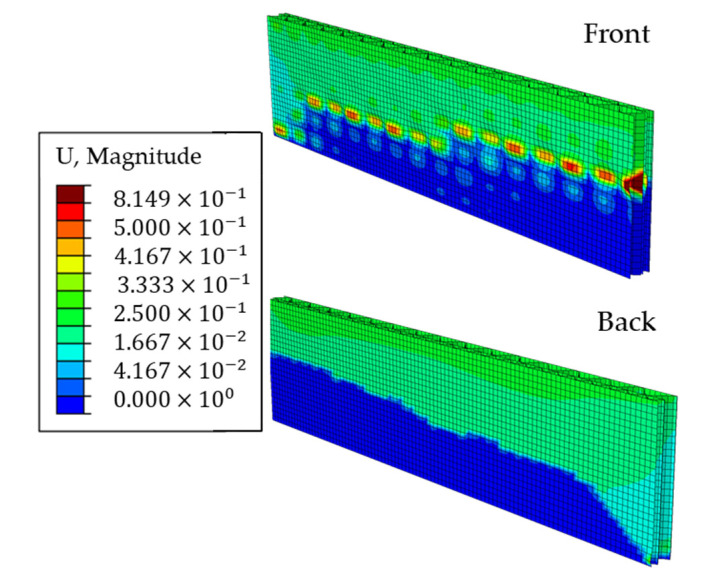
Displacements on both sides of the ECT sample in the last iteration of the nonlinear analysis.

The displacements on both sides of the ECT sample in the last iteration are shown in Figure 14, where the unloaded part of the sample is shown in blue.

Figure 13 and Figure 14 clearly show that the induced imperfections cause the first-mode buckling deformation of the sample and eventually the sample is damaged at about half its height. Numerical observations confirmed the experimental results, in which the correct failure mode in the ECT is the crush (crease) of the sample between its span, not the crush at the edges.

### 3.5. Compilation of All Results

**Figure 15:**
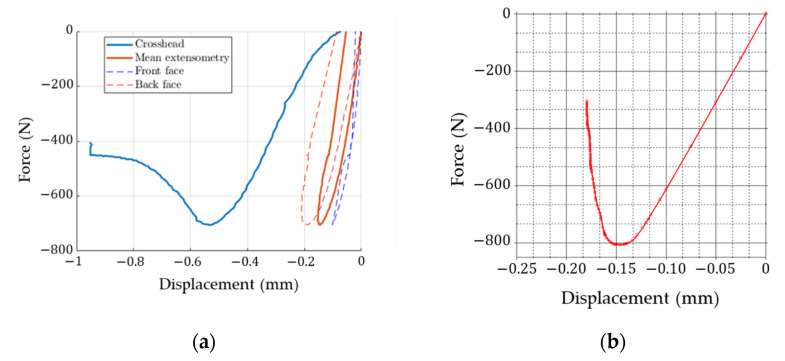
Experimental and numerical results: (**a**) load–displacement curves from experimental studies; (**b**) load–displacement curves from numerical studies.

Experimental results based on non-contact full-field displacement measurements and crosshead displacement (see Figure 15a) are presented here together with the results from various predictive models. Figure 15b shows the force-displacement curve obtained from a numerical full detailed 3D FE model.

**Figure 16:**
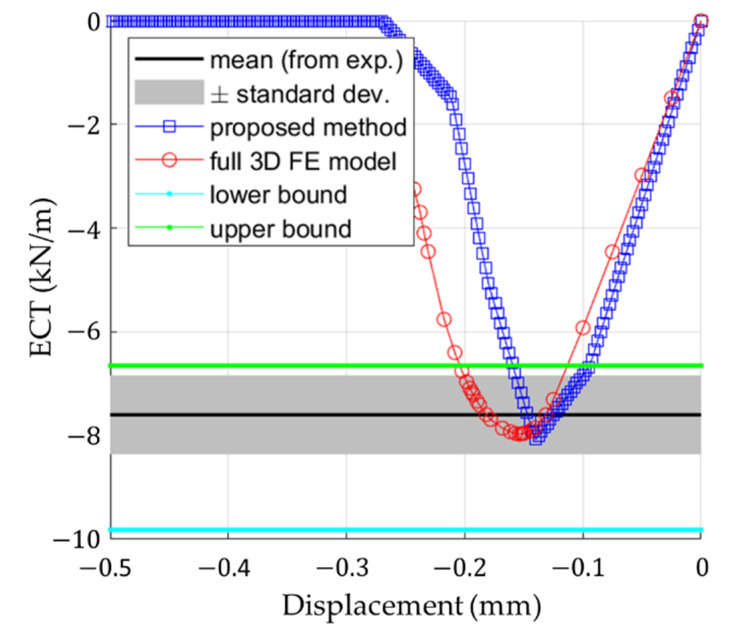
Summary of all results.

Figure 16 presents the summary of all results including the lower and upper bound of the analytical solutions. The lower bound can be computed by the formula:

(12) $$P_{min}=\epsilon_{min}\sum_{i=1}^{n}E_{CD}^{i}\alpha_{i},$$

where minimal strain, $\epsilon_{min}$ equals:

(13) $$\epsilon_{min}=\min\left(\frac{p_{max}^{i}}{E_{CD}^{i}}\right),$$

while the upper bound can be obtained from the equation:

Figure 16 also shows the results of the analytical method proposed here, as well as the experimental data (mean value ± one standard deviation) and results from the numerical validation model. Table 5 summarizes all measured and computed stiffnesses and compressive strengths in CD of the corrugated board 5EB650C3.

## 4. Discussion

The first and main observation (conclusion) to be drawn from this study is that the overall stiffness of a corrugated board sample cannot be determined from a typical ECT. This is clearly seen in Figure 10 and Figure 15a, where the slope of the force-displacement curve (with displacement measurement from the crosshead) gives about a three times less rigid response than when the displacement measurement is based on optical extensometry (Figure 15a). The use of non-contact measurement techniques makes it possible to correctly measure the displacements on the outer surfaces of the sample (excluding the edge areas of the sample and crosshead compliance). Therefore, in order to determine the stiffness of the corrugated board in the CD in the ECT, virtual extensometers are required.

The results summarized in Table 5 show that the analytical and numerical models give very similar values for the compressive stiffness compared to the measurement based on optical extensometry, while the stiffness value calculated from the displacements of the crossbars (on both machines) is three times smaller and cannot be treated as a representative value. The difference between the measured/calculated compressive stiffness does not exceed 10%. The compressive strength measured and calculated using both: (a) the numerical model and (b) the proposed analytical method differs by about 5% from the stated value of 7.6 kN/m. The results obtained with the analytical model are slightly higher than the measured values (stiffness: 9%, compressive strength: 6%). The full 3D FE model gives a slightly higher value of compressive strength (4.5%) and a slightly lower value of compressive stiffness (8%), which may be the result of introduced imperfections.

The second conclusion is that the proposed analytical formula for estimating stiffness and compressive strength appears to be very promising. It has the same accuracy as a full detailed 3D FE model (see Figure 15b and Figure 16) while being easier to implement and much faster to operate. Both analytical and numerical models can easily capture the compressive strength, ECT, and the overall stiffness of the corrugated board in the CD, as evidenced by the experimental results (see Figure 16). It is worth noting that the force-displacement curves from the optical measurement are in good agreement with the curves plotted by the predictive models (the maximum force appears between the displacement range: 0.1–0.2 mm, see Figure 15 and Figure 16).

**Figure 17:**
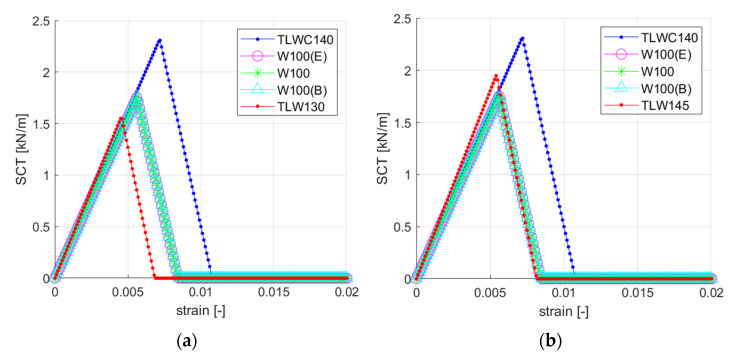
Stress-strain curves: (**a**) TLW120 exchanged with TLW130; (**b**) TLW120 exchanged with TLW145.

If one would like to optimize the design of the corrugated board by appropriate selection of solid boards for individual layers, it is enough to raise the basis weight of the weakest layer (in this case the TLW120 layer). By drawing the stress–strain curves of individual layers using Equations (3)–(6), it is easy to determine, which layer is the weakest. Figure 17 shows the constitutive curves of individual layers, taking into account the increase in the grammage of the TLW120 layers by 10% (Figure 17a) and 20% (Figure 17b).

Table 6 summarizes the simulation results where the basis weight of each of the layers was increased by 10 and 20 percent, respectively, and the effect of this change on the estimated edge crush resistance of the cross-section was checked using Equation (6). By far the biggest improvement is noted when the TLW120 layer is changed (strengthened). By increasing the basis weight of this layer by 10%, the ECT increases by 10.64%, and by increasing the basis weight by 20%, the ECT changes by as much as 24.33%.

## 5. Conclusions

This paper presents predictive models for the evaluation of compressive strength and stiffness of corrugated board in CD. The models proposed here and the obtained analytical and numerical results were compared with the experimental results. Good agreement in the obtained results was observed. The accuracy achieved with the full 3D FE model was within 95%, while the accuracy of the simplified analytical model was around 94%. Similar results were obtained by Perks et al. [12], who modeled different standards of the ECT test using the finite element method. However, the ECT prediction methods presented here (using both analytical and numerical models) are slightly more accurate than the results obtained by Parks et al. Only the optical measurement allows the correct drawing of load–displacement curves in the edge crush test. The use of crosshead displacement could not be used to calculate the stiffness of the corrugated board. In further research, further investigations on the use of full-field measurement methods (DIC) to estimate more material constants from the edge crush test are planned.

## Figures and Tables

**Table 1:** The geometrical features of both corrugated layers of 5EB650C3.

Wave (Flute)	Pitch [mm]	Height [mm]	Take-Up Ratio [–]
E	3.50	1.18	1.242
B	6.48	2.5	1.315

**Table 2:** The mechanical properties of individual layers of 5EB650C3.

Layer Name	Thickness [μm]	$E_{MD}$ [kN/m]	$E_{CD}$ [kN/m]	$SCT_{CD}$ [kN/m]
TLWC 140	180	725	323	2.32
W 100	160	886	328	1.76
TLW 120	170	907	313	1.81

**Table 3:** The mechanical properties of individual layers of 5EB650C3.

Layer Name	$E_{1}$ [MPa]	$E_{2}$ [MPa]	$\nu_{12}$ [–]	$G_{12}$ [MPa]	$G_{13}$ [MPa]	$G_{23}$ [MPa]
TLW 120	5669	2050	0.176	1319	103	59
W 100	5537	2050	0.209	1112	101	59
TLWC 140	4028	1794	0.196	1040	73	51

**Table 4:** The mechanical properties of individual layers of 5EB650C3.

Layer Name	b (mm)	L (m)	$SCT_{CD}$ (kN/m)	$P_{cr}$ (kN/m)
TLWC 140	3.50	25	2.32	4.212
W 100 (E)	2.17	25	1.76	9.573
W 100	-	25	1.76	-
W 100 (B)	4.26	25	1.76	2.444
TLW 120	6.48	25	1.81	1.237

**Table 5:** The measured/calculated compressive stiffness and strength in CD of the corrugated board 5EB650C3.

Test/Model	$ECT_{CD}$ (kN/m)	$E_{CD}$ (kN/m)
Producer specification	7.60	-
FEMat—crosshead	7.51	1991
Instron—crosshead	7.03	2142
Instron—opt. extensometry	7.03	6442
Numerical model	7.94	5920
Analytical model	8.08	7063

**Table 6:** The effect of improving individual corrugated board layers by 10 and 20 percent, respectively, on the changes in the ECT.

Reference		Single Layer Improved by 10%			Single Layer Improved by 20%		
Paperboard Symbol	ECT (kN/m)	Paperboard Symbol	ECT (kN/m)	Diff. (%)	Paperboard Symbol	ECT (kN/m)	Diff. (%)
TLWC 140	8.08	TLWC 155	8.261	2.24	TLWC 170	8.442	4.48
W 100 (E)	8.08	W 110 (E)	8.297	2.69	W 120 (E)	8.515	5.38
W 100	8.08	W 110	8.255	2.17	W 120	8.430	4.33
W 100 (B)	8.08	W 110 (B)	8.310	2.85	W 120 (B)	8.541	5.71
TLW 120	8.08	TLW 130	8.940	10.64	TLW 145	10.046	24.33

## Data Availability

The data presented in this study are available on request from the corresponding author.
